# Health care quality model of family physician program in Iran (mixed method)

**DOI:** 10.22088/cjim.13.4.666

**Published:** 2022

**Authors:** Alireza Heidarian Naeini, Ghahraman Mahmoudi, Jamshid Yazdani Charati

**Affiliations:** 1 Health Services Management, Islamic Azad University, Sari Branch, Sari, Iran; 2 Department of Epidemiology and Biostatistics, Mazandaran University of Medical Sciences, Sari, Iran

**Keywords:** Primary health care, Health care quality, Physicians, Family

## Abstract

**Background:**

There has been a growing international evolution of the role and purpose of quality improvement in primary health care. The present study aimed to develop a quality model of the Family Physician program in Iran.

**Methods:**

In the qualitative part of these mixed-method studies, grounded theory was used according to the systematic method of Strauss and Corbin. Semi-structured interviews were conducted with recipients and providers of Family Physician cares in the pilot provinces of Iran in 2020 to 2021 and continued until the theoretical saturation based on the purposive technique. The qualitative evaluation of the model was performed and approved. Structural equation modeling and Amos software were used to quantify the model.

**Results:**

The results of the structural equation analysis showed that the conceptual model of the research with chi-square test was 2.96 and RMSEA= 0.066, GFI=0.860 are well fitted. Structure, context, process, accountability, attitude, and empowerment factors directly and indirectly provide good predictors for the quality of care in the family physician program. The most important research findings in the field of quality improvement in the family physician’s cares included factors such as developing the attitude and vision of society, providers and policymakers in health subject and health needs, simultaneously corrections in all levels of the referral system, attention and adaptation to the context of society, developing the infrastructures and improving the related processes, systematic appraisal, and accountability and pay attention to the empowerment.

**Conclusion:**

To achieve the quality of care in the family physician program, we need change and development in our attitudes, context, infrastructures and processes, accountability and empowerment systems, and overall modification***.***

In almost all countries, the health care system undergoes reforms and changes to improve efficiency and effectiveness (1). The structure of health care systems and the coherence and coordination between affairs may determine the efficiency and the degree of achieving goals (2). Many countries have implemented family practice and a referral system based on level of care systems to achieve a universal health coverage. Family physician program (FPP) has been implemented as a part of macro policies in the health system (3). In the Middle East, FPP has been one of the health care policy priorities, even though specific local obstacles and hurdles have hindered a full and successful implementation of the program (4). Since 1985, Iran has established the primary health services network to respond to the health needs of individuals (5). In Iran, the FPP has been implemented in rural areas since 2006. 

Afterwards, the urban family medicine program was implemented in 2012 in Fars and Mazandaran provinces, unfortunately without a good understanding of the context, assessing capabilities and maturity level, cost management, and future state analysis (5-10). However, any policy plan in the implementation phase may face challenging issues. These challenges and problems in Iran led to a relative failure of the program at the urban level, which indicates unsolved basic problems. The results of some studies showed that after eight years of implementing family physician program in pilot provinces, what is implemented is far from the ideal FPP. There are requirements and standards that are not yet met (5-10). On the other hand, there is a growing international consensus regarding the impact of executive elements on the delivery of high-quality care. Still, the relationship between implementation and quality improvement is complicated (11). An understanding of the executive elements of any program is part of a broader concept of successful continuous quality improvement. Although, there are several surveys of executive challenges and problems of family medicine (5-10, 12), no executive model has been proposed that can be used to better implement family medicine services. The present research is focused on improving health care in FPP and identifying and developing a structural model of qualified health care in FPP. The main purpose is to develop a quality model for FPP in Iran.

## Methods

Through a mix methodology, the quantitative and the qualitative dimensions were covered including an in-depth investigation and a constructive synthesis of qualitative and quantitative data from the pilot provinces of the urban family physician project between 2020 and 2021. 

The study was carried out using a grounded theory approach for the design. The approach helps us with developing new theories (13). Grounded theory is a primarily qualitative methodology which in nature is inductive. The study does not try to test a specific hypothesis, but it rather tries to develop a hypothesis. Using this approach allows the researchers (professors and students of Mazandaran universities (not only consider which factors influence the quality of care in family medicine, but also understand the relationship among those factors. Through this, these factors can be organized as a cohesive theoretical model. Totally, 17 key informants were identified including recipients of family physician services, family physicians and health policy-makers, senior managers, and those fully informed about the family physician project for at least three years. They were selected via purposive and snowball sampling. Prior to the interview, the participants were presented with a letter of consent outlining the study plan. An interview guide was developed consisting of basic questions to clarify and expand on key themes. One of the questions was “What are the executive components affecting the quality of care in family medicine?” The Joint Commission definition of “quality of health care” was used including efficiency, effectiveness and evidence-based service, safety, respect and caring, timeliness, availability & accessibility, patient center, and continuity in care (14).

The Joint Commission is a United States-based organization that accredits health care organizations and programs and the international branch authorizes medical services from around the world (15). As the lead and main researcher, I gathered data through interviews from April to December 2020. The interviews lasted from 60 to 110 minutes. Face-to-face interviews were recorded on a portable MP3 recorder. Interviews were conducted by one researcher, and continued until theoretical saturation was reached. As a researcher knowledgeable in interview techniques and fluent in family physician program, I was aware that when interviewing my colleagues, I needed to try and remain neutral, setting aside my own views and reactions and to listen from a researcher’s perspective. 

 It was however difficult for me to be totally objective and set aside my personal experience, and thus taking an insider position. After each interview, the recorded voice would be transcribed verbatim. Then, the author would read the text for several times to familiarize himself with the text before coding. The codes and categories were extracted through induction, which was started with open coding and reading the text. The codes were then assigned. Categories were formed by systematic comparison and a member-check strategy to achieve an agreement about coding and increase viability. Comments were also added in the final analysis. Through this, the harmony of the result with experts’ opinions, beliefs and perception was ensure. Data gathering and analyzing were performed simultaneously. The concepts were formed following completion of coding and making sure of the precision. Data analysis was done using MAXQDA 18. Through prolonged engagement with data and experts for four months and performing several interviews, the researchers were able to assure data creditability. As check dependency, member check and peer check were used. To make sure of dependability, each abstract was provided to each expert throughout the interviews. Following the interviews, researchers listed the valuable themes and developed the primary theoretical models as follows:

**Figure 1:**
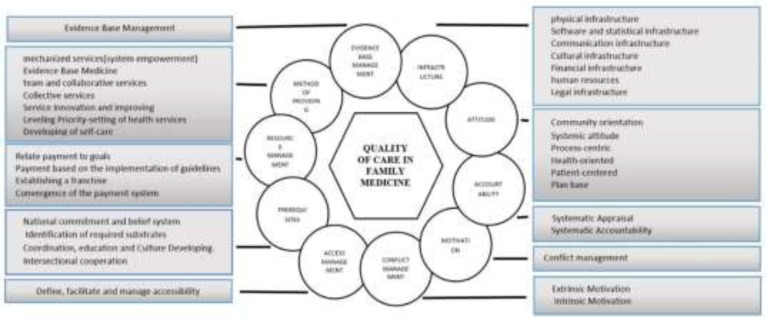
Initial theoretical model of quality of care in family physician programme

Extracted codes and categories and initial theoretical model of Health Care Quality of family physician program in Iran from the face-to-face interviews of 17 key informants (recipients and providers of family physician services) in response to key question of “What are the executive components affecting the quality of care in family medicine? that resulted in 10 themes and 35 subthemes.

**Expert consultation: **Then, based on the data collected in the previous levels, a questionnaire was designed. The questionnaire was finalized with 50 items and 11 parts: demographic characteristics (6 items); **prerequisites** (4 items); infrastructure (7 items); approaches (14 items); evaluation and accountability (3 items); **motivational** and supportive mechanism (1item); **resource** management (5 items); alignment of interests (1 item); evidence-based management (1 item); improve accessibility (1 item); method of providing services (7 items).

**Data Collection: **The study took three months from October 2020 to January 2021. Data gathering was done through the survey method. To randomly select the recipient of family medicine services in Mazandaran and Fars province, the sample and setting sections were used. Researchers first described the study to them throughout the study. After securing their consent to participate, the questionnaire was completed. The items of the questionnaire were designed based on Likert’s five-point scale (1 = strongly disagree, 5 = strongly agree). The tool was modified and finalized using feedback from eight experts and a pilot group of 30 experts. To examine the content validity quantitatively, the content validity ratio (CVR) and content validity index (CVI) was employed. The value of CVR was measured by reviewing the designed question of each item using a three-part spectrum (necessary, useful but not necessary, and not necessary). Based on Lawshe’s table, the items with CVR >83% as determined by 30 experts were selected as statistically significant items (p<0.05) and remained in the study. To examine CVI, Waltz, and Bausell measure was used so the items with CVI >0.7 remained in the tool.(16). The results supported good content validity of the tool.

**Respondents and questions: **337 clients took part in the study with a response rate of 95.2%. 22 questionnaires were not included in the study because of missed out information or being incomplete. Therefore, the effective rate of the tool was equal to 95.22%. The reason for some non-participation was lack of time or interest in the subject.Response to demographic characteristics of the respondents reveal that 188 (60%) are males and 127 (40%) are females. 93 (30%) of the respondents were in the age range of 21–30 years, 121 (38%) 31–40 years, 69 (22%) 41–50 years and only 42 (10%) were in the 51–60 years age range. 

The educational levels showed 8 (2%) with Medical Degree /Doctor of Philiosphy (PhD), 68 (22%) had Master’s degree, 136 (43%) with Bachelor’s degree, and 103 (33%) had Associate or lower degree holders. 59% of participants were residents in Fars and 41% in the province of Mazandaran. Most of the respondents completed the questionnaire within 25 min, with an average completion time of 27 min. Ethical approval for this study was obtained from Biomedical Research Ethics Committee of Islamic Azad University (IR.IAU.CHALUS.REC.1399.027).

**Data analysis: **With Cronbach’s alpha equal to 0.787, the credibility of the tool was supported. Based on factor analysis of the scale, the **Kaiser-Meyer-Olkin** (KMO) was equal to 0.759, so, there were several common factors among the variables. KMO is a statistical measure that indicates the proportion of variance in variables that might be caused by underlying factors. High values (close to 1.0) generally indicate that a factor analysis may be useful with data (17). Based on Bartlett’s spherical test, the χ2 value was 5086.748 (df = 946), p<0.001, which is indicative of common factors among the correlation matrices, so the scale can be subjected to factor analysis. In this way, the scale demonstrated a high structural validity.

**Exploratory factor analysis: **221 (70%) participants out of 315 were assigned to the exploratory factor analysis (EFA) group randomly. EFA is one of a family of multivariate statistical methods that attempts to identify the smallest number of hypothetical constructs (18). The collected data were used to examine possible scale structures through EFA. Taking into account the chance of high correlation between different factors, orthogonal rotation processing was done using the rotation method so each item was assigned with different factor loading in each common factor, which helped to determine the common factors. To make sure of differentiation of items, they were selected on their factor loading in each common factor. That is, items with a factor loading of 0.4 or higher remained to make sure of identification of the item, which also helped distinguish scale structure. Items that had a factor loading below 0.4 were removed. SPSS software Version 22 was used to perform Varimax rotation. Following EFA, six common factors were removed including 31 evaluation items (table 1).

**Table 1:** Exploratory factor analysis loadings of family physician quality model (exploratory factor analysis loadings of family physician quality model created by Categorize questionnaire questions through orthogonal Varimax rotation processing via SPSS software in which six common factors were extracted including 31 evaluation items)

	**EFA factors (n = 221)**					
**Item**	**factor1**	**factor2**	**factor3**	**factor4**	**factor5**	**factor6**
	**Processes**	**Attitude**	**Accountability**	**Structure**	**Context**	**Empowerment**
1a.Timely and appropriate payment	0.759					
2a.The definition, implementation and monitoring processes	0.756					
3a.Leveling Priority-setting of health services	0.751					
4a.Developing of self-care	0.708					
5a.Introducing Patient rights	0.704					
6a.Accountability of providers	0.686					
7a.team and collaborative services	0.657					
8a.Patient participation in care	0.603					
9a.Service Innovation and improving	0.578					
1e.Identification of required substrates		0.887				
2e.Considering of goals and priorities of health care providers		0.789				
3e.Culturally Competent Family Medicine		0.754				
4e.Coordination, education and Culture Developing.		0.731				
5e.Definition of the role and position of family physicians and health care providers		0.664				
1d.Collective services			0.781			
2d.Software and statistical infrastructure			0.682			
3d.Health-oriented education			0.644			
4d.physical infrastructure services			0.618			
5d.Welfare of clients			0.609			
6d.Communication infrastructure			0.609			
7d.sustainable resource allocation			0.579			
1b.National commitment and belief system				0.884		
2b.Systemic attitude				0.868		
3b.health-oriented approach of Stakeholder				0.745		
4b.Holistic performance				0.671		
1c.Law enforcement					0.858	
2c.Comprehensive and impartial regulatory structure					0.795	
3c.Accountability of officials					0.569	
1f.mechanized services (system empowerment)						0.668
2f.Evidence-based management						0.646
3f.empowerment of human resources						0.549

**Interpretation:** To interpret the data, the authors examined and determined which one of the variables was attributable to a factor and gave a name of the theme to that factor. Normally, each factor is loaded with two or three variables at least to give it a meaningful interpretation. Factor labeling is a theoretical, subjective, and inductive process. According to Henson and Roberts (2006), whether a latent factor is meaningful depends on the researcher’s definition (19). By conducting thorough and systematic factor analysis, we can isolate items with high loadings in the obtained pattern matrices. That is, it is an attempt to find the factors that explain the major part of the responses. If the researchers are satisfied with the factors, they will be operationalized and labeled descriptively. It is imperative that these labels or constructs be reflective of the theoretical and conceptual intent. According to table 1, the factor 1 by 9 items was named "Process”, factor 2 were named "Attitude"(4 items), the factor 3 was named "Accountability "(3 items), The factor 4 is called "Structure"(7 items), factor 5 was named "Context (5 items) and factor 6 by three items was named "Empowerment". Linear Regression was used to describe relationships between 6 latent values of model according to table 2.

**Table 2:** Regression coefficients of variables from service recipients’ perspective (latent values affecting the quality of care and regression coefficients of variables from service recipients perspective in F.P.P., T value, standard error and a p-value of correlations that represents a meaningful relationship between the variables (structure, accountability, attitude, context, process, empowerment)

**Latent values affecting the quality of care in F.P.P recipients perspective recipients**			**T**	**standard error**	**coefficient**	**P**
Structure		accountability	2/454	0/044	0/107	0/014
Accountability		Attitude	2/664	0/079	0/21	0/008
Attitude		empowerment	1/795	0/064	0/115	0/073
Context		Process	5/037	0/053	0/268	***
Accountability		Process	3/359	0/093	0/313	***
Empowerment		Process	-3/115	0/145	-0/452	0/002
Attitude		Process	5/177	0/078	0/406	***

**Confirmatory factor analysis: **Based on the remaining set of samples that had been randomly selected for EFA (100% of samples), confirmatory factor analysis (CFA), was carried out. CFA is a statistical technique used to verify the factor structure of a set of observed variables(18).

**Goodness of fit indices: **In this study, the model, which is obtained through field studies, should be consistent with the expected model in the real community. To examine the model fit, five indices were used including normalized fit index (NFI), the ratio of chi-square to a degree of freedom (x2/df), non-normalized fit index (NNFI), root mean square error of approximation (RMSEA), and comparative fit index (CFI). With NFI, NNFI, and CFI higher than 0.8, the close fit of a model to the data is supported. In addition, RMSEA<0.05 indicated a good fit (19). To check the internal consistency of potential factors, Cronbach’s alpha was used and the reliability would be acceptable if α ≥0.70 (p<0.05). Cronbach's alpha was calculated for each structure including process (0.86), context (0.81), infrastructure (0.88), attitude (0.91), accountability (0.91), and empowerment (0.89). Cronbach's alpha of the whole tool was 0.87. We performed EFA and CFA using AMOSE version 24. To improve the goodness-of-fit test (GOF), those questions, which had a factor load of less than 0.5, were deleted, and then software suggestions and feedback were implemented in the default model. The goodness-of-fit test is a statistical hypothesis test to see how well sample data fit a distribution from a population with a normal distribution(18). Negative suggestion, suggestions below 10 units, and suggestions which are not based on research literature were excluded. After applying the fit corrections, all fit indicators of the tree economic categories, namely, absolute and comparative fit index and economic fitness except RFI obtained the allowable limit; therefore, the modified measurement model has a good fit according to table 2. 

**Structural Model: **The structural model was implemented in Amos 24. After implementation in the software and consideration of the corrections, the final structural model of the research, which evaluates the relationship between latent values, was implemented as follows:

**Figure 2:**
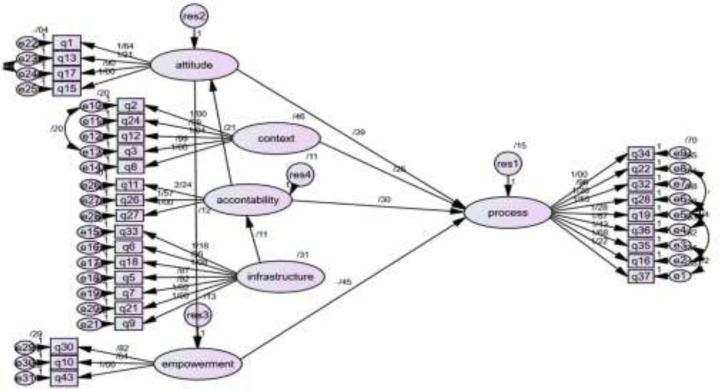
EFA and CFA and final structural model of care quality in family physician program implemented in Amos and SPSS software and consideration of the corrections. which evaluates the relationship between latent values obtained, obtained from the results of 315 questionnaires completed by recipients of family medicine services that resulted in 31 observed and 6 latent values

According to the results for model fit, the six-factor model showed the best fit index; The item combination of the six-factor model. The default, saturated and independence model fit indices with all data is according to table 3.

**Table 3:** Results of the confirmatory factor analysis (Results of the confirmatory factor analysis by Amos software, Goodness of fit indices, Reference value and Model performance (default, saturated and independence model) Which showed six-factor model the best fit index)

**Model performance**				**reference value**		**Goodness of fit indices**
**independence**	**saturated**	**default**				
2.961	2.416	3.246	X2 / df < 3		X2 (chi square)/ degrees of freedom	
0.066	0.067	0.085	RSMEA < 0.08		RSMEA (Root mean square error of approximation)	
0.728	0.729	0.673	PNFI > 0.5		PNFI (Parsimony Normed Fit Index)	
0.860	0.846	0.791	GFI > 0.8		GFI (Goodness-of-fit index)	
0.834	0.816	0.753	AGFI > 0.8		AGFI (Adjusted goodness-of-fit index)	
0.806	0.816	0.750	NFI > 0.8		NFI (Normed Fit Index)	
0.846	0.868	0.791	TLI > 0.8		TLI (Tucker Lewis index)	
0.861	0.882	0.811	CFI > 0.8		CFI (Comparative fit index)	
0.785	0.794	0.723	RFI > 0.8		RFI(Relative fit index)	
0.862	0.883	0.812	IFI > 0.8		IFI(Incremental fit index)	

## Discussion

The model developed indicates that there are six predictors of the quality of family practice services. In other words, they must be considered for the quality of services provided through the family medicine program. These components include: attitude, accountability, context, empowerment and process. In addition to being able to independently express the quality of services in family medicine, these components can also significantly impact each other. For example, Attitude, Accountability, Context, Empowerment have an effect on the Process and Infrastructure on Accountability, Attitude on Empowerment and Accountability on Attitude. 

There are various models of service quality; however, these models did not thoroughly address approaches or attitudes as an influential component. However, the results of the current paper showed that attitude is an important component for achieving quality services in the family medicine. The health-oriented approach, both in policy making and in education, is one of the most important components affecting the quality of family medicine services. This means that the approach or attitude affect macro policies, processes, and outcomes. Shifting the method of providing services in the primary care from treatment-centered to health-centered requires changing the approach of policy makers, the education system, physicians, as well as changing the approach and attitude of society towards health. Furthermore, national determination and the authorities’ belief and will are among the most important influential components. It cannot be expected that the family medicine plan is able to guarantee the quality of its services in the absence of the above factors. Implementing reforms at primary care is not possible without considering specialized levels and specialized providers. These reforms in the family medicine must comprehensively and systematically cover all levels of health care system at the same time to achieve the right result. This finding is consistent with other studies (20-24).

As for empowerment, many papers merely take into account the employee empowerment. However, the current paper showed that the empowerment, in addition to providers, includes a wider range of policy makers, managers, and planners, even the care recipients’ empowerment to develop self-care, as well as the system and structural empowerment. The result is consistent with other studies conducted by Yoshida(21), Wanjau (24) and Farshad (25). The current paper showed that if a plan does not comply with the context of society habits, culture, and customs, it will definitely not lead to good service quality results. Therefore, the FPP needs to have the necessary flexibility to adapt to the context of society. Thus, either the FPP should be adapted to the treatment-oriented context of the country or the country's health culture should be modified simultaneously with the implementation of family medicine. There is a need to take into account the role of existing media and processes as well as paying attention to the power sources and interests of beneficiaries (22, 24-30). Other main categories that require closer attention in the quality of family physicians care include supervision, control, and accountability. Monitoring, auditing, and accounting mechanisms, facilitate service assessment, as well as the formulation of laws and regulations. More importantly, the implementation of the law through existing regulatory levers, including the organization of the medical system and medical universities are among the most important categories to address in achieving the family medicine quality services. The authorities, providers, and even the community’s accountability to the FPP are effective in achieving quality of care. These findings are consistent with previous studies (24, 27). 

Infrastructures such as physical, financial, cultural, communicative, statistical, software and educational infrastructures and provision of sustainable resources are also important. Optimal allocation of sustainable and dedicated resources and timely and adequate payment to service providers are effective in the quality of care of family medicine. The other finding of the current paper is the relationship between infrastructure and accountability. This is because the process, statistical, and systemic infrastructures facilitate the accountability process. Therefore, the necessary infrastructure must be provided to increase accountability and better enforce the rules. These findings are consistent with other studies (23-27, 31, 32). The importance of processes and its impact on service quality has recognized many quality models. Many effective components, in addition to direct impact, also indirectly increase the care quality through changes in processes. In the proposed model, these components affect the attitude, context, accountability, empowerment and processes. The main processes include the financial and psychological motivation of stakeholders, maintaining, aligning and adjusting stakeholders’ interests, linking pay to performance, cross-sectorial cooperation, convergence of payment systems, physician-patient interaction, effective resource allocation, accessibility improvement, collaboration among providers, improvement of patients’ participation and cooperation, and using new methods of providing services such as telemedicine and home care. These findings consisted with other studies (24, 25, 33). 

In conclusion, to achieve the quality of care in FPP, there is a need to change and improve our attitudes, context, infrastructures and processes, accountability, empowerment systems, along with general modifications.

## References

[1] Frenk J (1994). Dimensions of health system reform. Health Policy.

[2] Hofmarcher MM,, Oxley H, Rusticelli E mproved health system performance through better care coordination. IDEAS 2007.

[3] Organization WH (2014). Conceptual and strategic approach to family practice: towards universal health coverage through family practice in the Eastern Mediterranean Region.

[4] Abyad A, Al-Baho AK, Unluoglu I, Tarawneh M, Hilfy T (2007). Development of family medicine in the Middle East. Fam Med.

[5] Behzadifar M, Behzadifar M, Heidarvand S (2018). The challenges of the family physician policy in Iran: a systematic review and meta-synthesis of qualitative researches. Fam Prac.

[6] Eskandari M, Abbaszadeh A, Borhani F (2013). Barriers of referral system to health care provision in rural societies in Iran. J Caring Sci.

[7] Heydari MR, Kalateh Sadati A, Bagheri Lankarani K (2017). The evaluation of urban community health centers in relation to family physician and primary health care in southern Iran. Iran J Public Health.

[8] Nasrollahpour Shirvani SD, Mikanik E, Ashrafian Amiri H (2014). Evaluation of the referral system situation in family physician program in northern provinces of Iran: 2012-2013. J Mazandaran Univ Med Sci.

[9] Janati A, Maleki Mr, Gholizadeh M, Narimani M, Vakili S (2010). Assessing the strengths & weaknesses of family physician program. Knowledge Health.

[10] Golalizadeh E, Moosazadeh M, Amiresmaili M, Ahangar N (2012). Challenges in second level of referral system in family physician plan: a qualitative research. J Med Council.

[11] Crossland L, Janamian T, Jackson CL (2014). Key elements of high‐quality practice organisation in primary health care: a systematic review. Med J Aust.

[12] NASR ESS, Raeisi P, Motlagh M, Kabir M, Ashrafian AH (2010). Evaluation of the performance of referral system in family physician program in Iran University of Medical Sciences: 2009. Hakim Res J.

[13] Corbin J, Strauss A (2014). Basics of qualitative research: Techniques and procedures for developing grounded theory: Sage Publications.

[14] da Costa FA, Van Mil JF, Alvarez-Risco A (2019). The pharmacist guide to implementing pharmaceutical care. Can J Hosp Pharm.

[15] Karaarslan I (2009). Joint Commission on International Accreditation workshop: Planning, development and provision of laboratory services. Clin Biochem.

[16] Wilson FR, Pan W, Schumsky DA (2012). Recalculation of the critical values for Lawshe’s content validity ratio Measurement and evaluation in counseling and development. APP PsycNet.

[17] Trujillo-Ortiz A Kaiser-Meyer-Olkin measure of sampling adequacy. A MATLAB file. KMO, MathWork 2006.

[18] Watkins MW (2018). Exploratory factor analysis: A guide to best practice. J Black Psychol.

[19] Williams B, Onsman A, Brown T (2010). Exploratory factor analysis: A five-step guide for novices. Aust J Paramed.

[20] Cleary SM, Molyneux S, Gilson L (2013). Resources, attitudes and culture: an understanding of the factors that influence the functioning of accountability mechanisms in primary health care settings. BMC Health Serv Res.

[21] Yoshida K (2011). Fundamental approach to quality improvement of Japan society of health evaluation and promotion. Health Evalation Promotion.

[22] Yazdani S, Akbarilakeh M (2017). Which Health Cares Are Related to the Family Physician? A Critical Interpretive Synthesis of Literature. Iranian J Public Health.

[23] Mosadeghrad AM (2014). Factors affecting medical service quality. Iran J Public Health.

[24] Wanjau KN, Muiruri BW, Ayodo E (2012). Factors affecting provision of service quality in the public health sector: A case of Kenyatta national hospital. Semantic Scholar.

[25] Tavakoli F, Nasiripour AA, Riahi L, Mahmoudi M (2020). Design of a model for management of referral system in the Iranian urban family physician program. Iran J Public Health.

[26] Kaplan HC, Brady PW, Dritz MC (2010). The influence of context on quality improvement success in health care: a systematic review of the literature. Milbank Q.

[27] Iyoha FO, Oyerinde D (2010). Accounting infrastructure and accountability in the management of public expenditure in developing countries: A focus on Nigeria. Crit Perspectives Accounting.

[28] Mainz J, Kristensen S, Bartels P (2015). Quality improvement and accountability in the Danish health care system. Int J Qual Health Care.

[29] Øvretveit J (2014). Perspectives on context How does context affect quality improvement. London: The Health Foundation.

[30] Chassin MR, Loeb JM, Schmaltz SP, Wachter RM (2010). Accountability measures—using measurement to promote quality improvement. N Engl J Med.

[31] Bossen C, Piras EM (2020). Introduction to the special issue on ‘information infrastructures in healthcare: governance, quality improvement and service efficiency’. Computer Supported Cooperative Work (CSCW).

[32] Kironde S, Kahirimbanyib M (2002). Community participation in primary health care (PHC) programmes: lessons from tuberculosis treatment delivery in South Africa. Afr Health Sci.

[33] Ojo OS, Malomo SO, Egunjobi AO, Jimoh A, Olowere MO (2018). Quality of primary care physicians’ communication of diabetes self-management during medical encounters with persons with diabetes mellitus in a resource-poor country. South Afr Fam Prac.

